# Supplementary material for: Pyruvate Dehydrogenase Kinase 1 inhibition mediated oxidative phosphorylation enhancement in cartilage promotes osteoarthritis progression
Source: BMC Musculoskelet Disord. 2023 Jul 20;24:597. doi: 10.1186/s12891-023-06585-6 (PMC10357736; doi:10.1186/s12891-023-06585-6)
Supplement: Supplementary file 1 — Additional file 1. [file 12891_2023_6585_MOESM1_ESM.zip › MF.Gsea.1653627316242/GOMF_PHOSPHATIDYLINOSITOL_BISPHOSPHATE_PHOSPHATASE_ACTIVITY.html]

Details for gene set GOMF\_PHOSPHATIDYLINOSITOL\_BISPHOSPHATE\_PHOSPHATASE\_ACTIVITY[GSEA]

|  || Dataset | 55457\_collapsed\_to\_symbols.cls.cls #OA\_versus\_normal.cls.cls #OA\_versus\_normal\_repos |
| Phenotype | cls.cls#OA\_versus\_normal\_repos |
| Upregulated in class | normal |
| GeneSet | GOMF\_PHOSPHATIDYLINOSITOL\_BISPHOSPHATE\_PHOSPHATASE\_ACTIVITY |
| Enrichment Score (ES) | -0.56333685 |
| Normalized Enrichment Score (NES) | -1.7803642 |
| Nominal p-value | 0.0 |
| FDR q-value | 0.071555234 |
| FWER p-Value | 0.62 |
Table: GSEA Results Summary

  

Fig 1: Enrichment plot: GOMF\_PHOSPHATIDYLINOSITOL\_BISPHOSPHATE\_PHOSPHATASE\_ACTIVITY      
 Profile of the Running ES Score & Positions of GeneSet Members on the Rank Ordered List

  

| SYMBOL | TITLE | RANK IN GENE LIST | RANK METRIC SCORE | RUNNING ES | CORE ENRICHMENT || 1 | FIG4 | FIG4 phosphoinositide 5-phosphatase [Source:HGNC Symbol;Acc:HGNC:16873] | 617 | 0.618 | 0.0527 | No |
| 2 | INPP5D | inositol polyphosphate-5-phosphatase D [Source:HGNC Symbol;Acc:HGNC:6079] | 3085 | 0.241 | -0.0995 | No |
| 3 | MTMR7 | myotubularin related protein 7 [Source:HGNC Symbol;Acc:HGNC:7454] | 4432 | 0.119 | -0.1847 | No |
| 4 | INPP4A | inositol polyphosphate-4-phosphatase type I A [Source:HGNC Symbol;Acc:HGNC:6074] | 5274 | 0.050 | -0.2419 | No |
| 5 | MTMR14 | myotubularin related protein 14 [Source:HGNC Symbol;Acc:HGNC:26190] | 5458 | 0.037 | -0.2501 | No |
| 6 | MTM1 | myotubularin 1 [Source:HGNC Symbol;Acc:HGNC:7448] | 5768 | 0.010 | -0.2724 | No |
| 7 | INPP5J | inositol polyphosphate-5-phosphatase J [Source:HGNC Symbol;Acc:HGNC:8956] | 6009 | -0.005 | -0.2902 | No |
| 8 | MTMR2 | myotubularin related protein 2 [Source:HGNC Symbol;Acc:HGNC:7450] | 6189 | -0.019 | -0.3010 | No |
| 9 | INPP4B | inositol polyphosphate-4-phosphatase type II B [Source:HGNC Symbol;Acc:HGNC:6075] | 6284 | -0.026 | -0.3040 | No |
| 10 | SYNJ2 | synaptojanin 2 [Source:HGNC Symbol;Acc:HGNC:11504] | 6358 | -0.032 | -0.3044 | No |
| 11 | MTMR4 | myotubularin related protein 4 [Source:HGNC Symbol;Acc:HGNC:7452] | 6899 | -0.072 | -0.3346 | No |
| 12 | OCRL | OCRL inositol polyphosphate-5-phosphatase [Source:HGNC Symbol;Acc:HGNC:8108] | 7319 | -0.103 | -0.3503 | No |
| 13 | MTMR1 | myotubularin related protein 1 [Source:HGNC Symbol;Acc:HGNC:7449] | 8102 | -0.163 | -0.3844 | No |
| 14 | INPP5K | inositol polyphosphate-5-phosphatase K [Source:HGNC Symbol;Acc:HGNC:33882] | 8103 | -0.163 | -0.3578 | No |
| 15 | MTMR3 | myotubularin related protein 3 [Source:HGNC Symbol;Acc:HGNC:7451] | 10751 | -0.399 | -0.4984 | Yes |
| 16 | MTMR8 | myotubularin related protein 8 [Source:HGNC Symbol;Acc:HGNC:16825] | 10991 | -0.429 | -0.4471 | Yes |
| 17 | MTMR6 | myotubularin related protein 6 [Source:HGNC Symbol;Acc:HGNC:7453] | 11085 | -0.442 | -0.3823 | Yes |
| 18 | SYNJ1 | synaptojanin 1 [Source:HGNC Symbol;Acc:HGNC:11503] | 11577 | -0.512 | -0.3370 | Yes |
| 19 | PTEN | phosphatase and tensin homolog [Source:HGNC Symbol;Acc:HGNC:9588] | 11836 | -0.556 | -0.2665 | Yes |
| 20 | INPP5E | inositol polyphosphate-5-phosphatase E [Source:HGNC Symbol;Acc:HGNC:21474] | 11882 | -0.568 | -0.1775 | Yes |
| 21 | PIKFYVE | "phosphoinositide kinase, FYVE-type zinc finger containing [Source:HGNC Symbol;Acc:HGNC:23785]" | 12347 | -0.690 | -0.1011 | Yes |
| 22 | INPP5B | inositol polyphosphate-5-phosphatase B [Source:HGNC Symbol;Acc:HGNC:6077] | 12723 | -0.884 | 0.0137 | Yes |
Table: GSEA details [plain text format]

  

Fig 2: GOMF\_PHOSPHATIDYLINOSITOL\_BISPHOSPHATE\_PHOSPHATASE\_ACTIVITY      
 Blue-Pink O' Gram in the Space of the Analyzed GeneSet

  

Fig 3: GOMF\_PHOSPHATIDYLINOSITOL\_BISPHOSPHATE\_PHOSPHATASE\_ACTIVITY: Random ES distribution      
 Gene set null distribution of ES for **GOMF\_PHOSPHATIDYLINOSITOL\_BISPHOSPHATE\_PHOSPHATASE\_ACTIVITY**
